# The heme and radical scavenger α_1_-microglobulin (A1M) confers early protection of the immature brain following preterm intraventricular hemorrhage

**DOI:** 10.1186/s12974-019-1486-4

**Published:** 2019-06-07

**Authors:** Olga Romantsik, Alex Adusei Agyemang, Snjolaug Sveinsdóttir, Sigurbjörg Rutardóttir, Bo Holmqvist, Magnus Cinthio, Mattias Mörgelin, Gulcin Gumus, Helena Karlsson, Stefan R. Hansson, Bo Åkerström, David Ley, Magnus Gram

**Affiliations:** 1 Pediatrics, Department of Clinical Sciences Lund, Lund University, Lund, Sweden; 2 Infection Medicine, Department of Clinical Sciences Lund, Lund University, Lund, Sweden; 3 ImaGene-iT AB, Medicon Village, Lund, Sweden; 4 Department of Electrical Measurements, Lund University, Lund, Sweden; 5 Fetal i+D Fetal Medicine Research Center, BCNatal Barcelona Center for Maternal-Fetal and Neonatal Medicine, University of Barcelona, Barcelona, Spain; 6 A1M Pharma AB, Lund, Sweden; 7 Obstetrics and Gynecology, Department of Clinical Sciences Lund, Lund University, Lund, Sweden

**Keywords:** Intraventricular hemorrhage, Hemoglobin, α_1_-microglobulin, Oxidative stress, Damage to the immature brain

## Abstract

**Background:**

Germinal matrix intraventricular hemorrhage (GM-IVH) is associated with cerebro-cerebellar damage in very preterm infants, leading to neurodevelopmental impairment. Penetration, from the intraventricular space, of extravasated red blood cells and extracellular hemoglobin (Hb), to the periventricular parenchyma and the cerebellum has been shown to be causal in the development of brain injury following GM-IVH. Furthermore, the damage has been described to be associated with the cytotoxic nature of extracellular Hb-metabolites. To date, there is no therapy available to prevent infants from developing either hydrocephalus or serious neurological disability. Mechanisms previously described to cause brain damage following GM-IVH, i.e., oxidative stress and Hb-metabolite toxicity, suggest that the free radical and heme scavenger α_1_-microglobulin (A1M) may constitute a potential neuroprotective intervention.

**Methods:**

Using a preterm rabbit pup model of IVH, where IVH was induced shortly after birth in pups delivered by cesarean section at E29 (3 days prior to term), we investigated the brain distribution of recombinant A1M (rA1M) following intracerebroventricular (i.c.v.) administration at 24 h post-IVH induction. Further, short-term functional protection of i.c.v.-administered human A1M (hA1M) following IVH in the preterm rabbit pup model was evaluated.

**Results:**

Following i.c.v. administration, rA1M was distributed in periventricular white matter regions, throughout the fore- and midbrain and extending to the cerebellum. The regional distribution of rA1M was accompanied by a high co-existence of positive staining for extracellular Hb.

Administration of i.c.v.-injected hA1M was associated with decreased structural tissue and mitochondrial damage and with reduced mRNA expression for proinflammatory and inflammatory signaling-related genes induced by IVH in periventricular brain tissue.

**Conclusions:**

The results of this study indicate that rA1M/hA1M is a potential candidate for neuroprotective treatment following preterm IVH.

**Electronic supplementary material:**

The online version of this article (10.1186/s12974-019-1486-4) contains supplementary material, which is available to authorized users.

## Introduction

Germinal matrix intraventricular hemorrhage (GM-IVH) is a frequent complication of prematurity, which occurs in 25% of very low birth weight (VLBW) infants [1, 2]. Despite a remarkable improvement in modern perinatal care, leading to a significant decrease in overall incidence of GM-IVH in preterm infants from 50% in the late 1970s to the current 15–28% [3–7], the absolute number of infants with GM-IVH remains high due to increased survival of extremely premature infants born below 28 + 0 weeks of gestation [8–11]. Of note, 45% of preterm infants with a birth weight below 1000 g are affected by some degree of GM-IVH and 35% of these lesions are severe [12–14]. Approximately 50% of survivors with GM-IVH develop cerebral palsy, mental retardation, post-hemorrhagic ventricular dilatation (PHVD), or a combination of these conditions [15]; and around a quarter of non-disabled survivors develop psychiatric disorders and executive function problems [16–18]. Importantly, from data presented in a recent systematic review and meta-analysis it seems that even a GM hemorrhage without apparent brain parenchyma damage has long-term consequences on neurodevelopmental outcome [19].

The etiology of GM-IVH is multifactorial, complex, and heterogeneous. It has been suggested that the hemorrhage originates in the GM, and with the further rupture of ventricular ependyma it evolves into IVH [20]. Gram et al. have shown that deposition of extravasated blood in the intraventricular space is followed by lysis of the red blood cells (RBC) resulting in a subsequent release of extracellular hemoglobin (Hb) into the cerebrospinal fluid (CSF) [21]. Once Hb escapes from the intra-erythrocyte compartment, it is highly reactive and oxidized from ferrous Hb (Fe^2+^, denoted oxyHb) to ferric (Fe^3+^, denoted metHb) [21, 22]. MetHb readily releases the heme group [23]. Free heme, iron, and reactive oxygen species (ROS) are redox reactive and can damage lipids, proteins, and DNA through oxidative modification, cross-linking, and fragmentation [24]. Moreover, heme is lipophilic and may intercalate cell membranes, resulting in cytolytic effects [25]. Using a preterm rabbit pup model of IVH, it has been observed that extracellular Hb leads to structural damage of the choroid plexus ependyma already at 24 h after IVH and causes severe cellular disintegration with loss of normal villous morphology and signs of cellular apoptosis/necrosis at 72 h after IVH [21, 26]. It has been shown that following GM-IVH, there is a wide distribution of extracellular Hb in periventricular white matter [27], an area rich in pre-oligodendrocytes. Pre-oligodendrocytes are highly vulnerable to oxidative stress, inflammation, and hypoxic-ischemic events [28, 29]. In addition, extravasation of RBCs and Hb into CSF have been shown to result in deposition of Hb-metabolites on the cerebellar surface, leading to alteration of normal development of the cerebellar cortex and its related functions [30–32]. Thus, it seems that RBC rupture and release of extracellular Hb may be central in the development of irreversible brain damage following GM-IVH.

Currently, GM-IVH is neither preventable nor treatable and hence there is a clear requirement for treatment strategies leading to prevention or reducing of neurologic consequences. Considering the potential causal role of extracellular Hb-metabolites in the development of brain damage following preterm GM-IVH, some Hb-, heme- and ROS-neutralizing systems have been investigated [26, 33, 34].

In this study, we evaluate the heme- and radical scavenger α_1_-microglobulin (A1M), a potent tissue-protective protein that has been shown to confer its effects through heme binding, reductase activity, radical scavenging, and binding to mitochondria [35, 36]. Furthermore, it has been demonstrated that mitochondrial uptake of A1M in early stages of cell death results in inhibition of heme- and ROS-induced mitochondrial swelling [37]. To date, a recombinant human A1M (rA1M) has been developed [38, 39] and it has been shown to be functionally equivalent to endogenous A1M derived from human plasma (hA1M, [35, 40, 41]). Here, we report that following intracerebroventricular (i.c.v.) administration, in preterm rabbit pups with IVH, rA1M is widely distributed within the periventricular white matter extending to the white matter of the cerebellum, within 72 h. Furthermore, i.c.v. administration of hA1M was found to confer early functional protection of the immature brain, evaluated at 24 and 72 h after hemorrhage.

## Materials and methods

### Animals

This study was approved by the Swedish Animal Ethics Committee in Lund. We used the well-established preterm rabbit pup model of glycerol-induced IVH as previously described [42]. Briefly, the experiments were performed on preterm rabbit pups, 29 pups (27 females and 2 males) in the distribution studies and 55 pups (sex was not determined) in the functional studies, delivered via cesarean section after the does were anesthetized with intravenous (i.v.) propofol (5 mg/kg, Primen Pharmaceuticals Oy, Helsinki, Finland) on day 29 (term 31–32 days). A half-breed between New Zealand White and Lop was used (Christer Månsson, Löberöd, Sweden). After delivery, the pups were dried and placed in an infant incubator set to a temperature of 32 °C and ambient humidity (which was achieved by the placement of a warm water (~32 °C) container within the incubator). At approx. 1–2 h of age, the pups were weighed, marked, and hand-fed with kitten milk formula (100 ml/kg/day, KMR, PetAg Inc., Hampshire, IL, USA) using a 3.5 French feeding tube (Vygon, Ecouven, France) and fed every 12 h. The administered amount of milk was increased every 24 h by 25 ml/kg/day. At approx. 2–3 h of age, the pups were injected intraperitoneally (i.p.) with 50% (*v*/*v*) sterile glycerol (6.5 g/kg; Teknova, Hollister, CA, USA) to induce IVH. Ultrasound imaging of the brain was performed at 6, 18, 24, 48, and 72 h of age to grade the severity of the IVH using the VisualSonics Vevo 2100 (VisualSonics Inc., ON, Canada) with a MS-550D 40 MHz transducer. Pups with severe IVH as determined by ultrasound were assigned to the IVH group, and those without detectable IVH at all time-points were used as controls. Measurements of ventricular size for assessment of PHVD were obtained at the level of the midseptal nucleus in a coronal view at 18, 24, 48, and 72 h of age. Each ventricle was measured horizontally from the midbrain plane to the lateral wall of the ventricle. Reproducibility and accuracy of ventricular measurements in this animal model using high-frequency ultrasound have been described previously [42].

### Experimental setup recombinant A1M (rA1M) distribution study

**Fig. 1:**
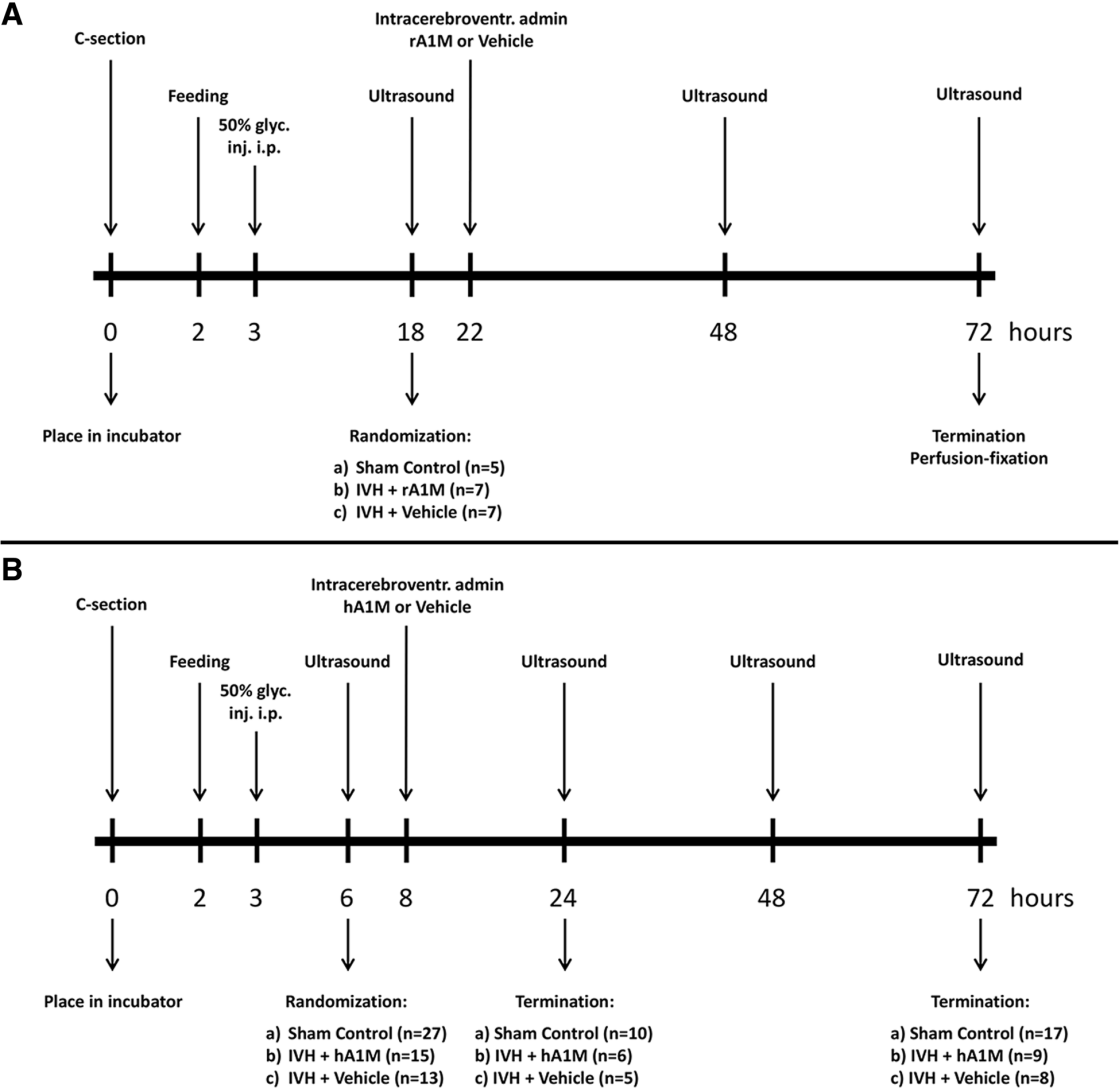
Schematic summary of the experimental procedure. The following steps were undertaken in both studies: Preterm delivery of rabbit pups via cesarean section on E29 (0 h); induction of IVH by i.p. administration of 50% (v/v) sterile glycerol (6.5 g/kg) at approx. 3 h of age; detection of IVH by high-frequency ultrasound. Feeding and verification of IVH, by high-frequency ultrasound, were performed throughout the study. For details about each step, see the “Materials and methods” section. **a** rA1M distribution. Randomization into study groups was done at approx. 18 h of age; i.c.v. administration of rA1M/Vehicle solution at approx. 22 h of age; termination and perfusion fixation of pups at 72 h of age. **b** hA1M functional protection. Randomization into study groups was done at approx. 6 h of age; i.c.v. administration of hA1M/Vehicle solution at approx. 8 h of age; termination of pups at 24 and 72 h of age

Following the brain ultrasound at 18 h of age, pups with IVH (presence of blood within distended lateral ventricles and no sign of parenchymal involvement) were randomized into one of the following two groups: IVH + rA1M (*n* = 7) or IVH + Vehicle (*n* = 7). Pups designated to either group were injected i.c.v. under ultrasound-guidance at approx. 22 h of age with either 25 μl of human recombinant A1M (rA1M, 9.4 mg/ml, A1M Pharma AB, Lund, Sweden) or 25 μl of vehicle solution (10 mM Tris-HCl pH 8.0, 0.125 M NaCl, A1M Pharma AB, Lund, Sweden), using 27 G Hamilton syringes (Hamilton Robotics, Reno, NV, USA). For these procedures the rabbit pups were gently fixated on a pre-heated thermostat-controlled platform at 39 °C in a prone position with the probe hand-held by one of the investigators and another investigator performed the needle-insertion under ultrasound guidance. The procedure was performed without sedation of the rabbit pups. The efficacy and accuracy of this method has previously been described [42]. Animals with no detectable IVH at all time-points on cranial ultrasound were used as controls (Sham Control, *n* = 5). The animals were euthanized at 72 h of age (corresponding to term-equivalent postnatal day 0). An overview of the study design is shown in Fig. 1a.

### Tissue collection and processing for histology and immunolabeling

Following sedation with i.p. ketamine administration (35 mg/kg, Intervet International B.V, Boxmeer, The Netherlands) and isoflurane inhalation (1000 mg/g, Virbac Carros, France), rabbit pups were euthanized, and perfusion fixation of the brain with phosphate buffer saline (PBS, pH 7.4, containing 0.01% of heparin) and freshly prepared 4% paraformaldehyde (PFA, VWR Chemicals, Leuven, Belgium, buffered with PBS, pH 7.4) was performed. After fixation, the brains were dissected out from the skull and post-fixed by immersion in 4% PFA. A change to fresh PFA was performed after 6–8 h and brains were then immersed in PFA for a total of 48 h, at 4 °C. The brains were rinsed in PBS (2 × 20 min) and processed for cryoprotection, by equilibration at 4 °C, first in 10% sucrose (Nordic Sugar AB, Arlöv, Sweden, diluted in PBS) and then in 20% sucrose (diluted in PBS), for a total of 16 h. The brains were divided in two parts, one containing the fore- and midbrain and the second the hindbrain and cerebellum. The brains were embedded in cryomolds with TissueTec (OCT, Sakura, Japan) and were frozen at around − 60 °C.

For histochemistry, a total of 11 brains (Sham Control, *n* = 3; IVH + rA1M, *n* = 5; IVH + Vehicle, *n* = 3) were used. Frontal serial cryosections, 12-μm thick, were collected on microscope slides. Series of adjacent sections for each brain were collected from four levels of the brain: forebrain, rostral midbrain, central midbrain, and caudal midbrain, used for the single- or double-labeling as described below. The sections thereby included the major parts of the ventricular system from the forebrain to the caudal midbrain, the surrounding germinal matrix, and the neurogenetic regions. Sections were stored at − 20 °C until used for the labeling (as described below).

Six rabbit pup brains (two from each experimental group) were paraffin embedded. After fixation (as described above) brains were dehydrated in a graded alcohol series ended with 100% xylen (Histolab, Gothenburg, Sweden), followed by infiltration in 100% paraffin (Histolab) and embedding in paraffin blocks. Prior to antibody staining for immunohistochemistry (IHC), the sections were rehydrated, followed by heat-induced antigen retrieval at 90–95 °C for 20 min in citric acid buffer (pH 6.0, 0.2% Triton-X 100, AppliChem, Darmstadt, Germany).

### Peroxidase Histochemistry

**Fig. 2:**
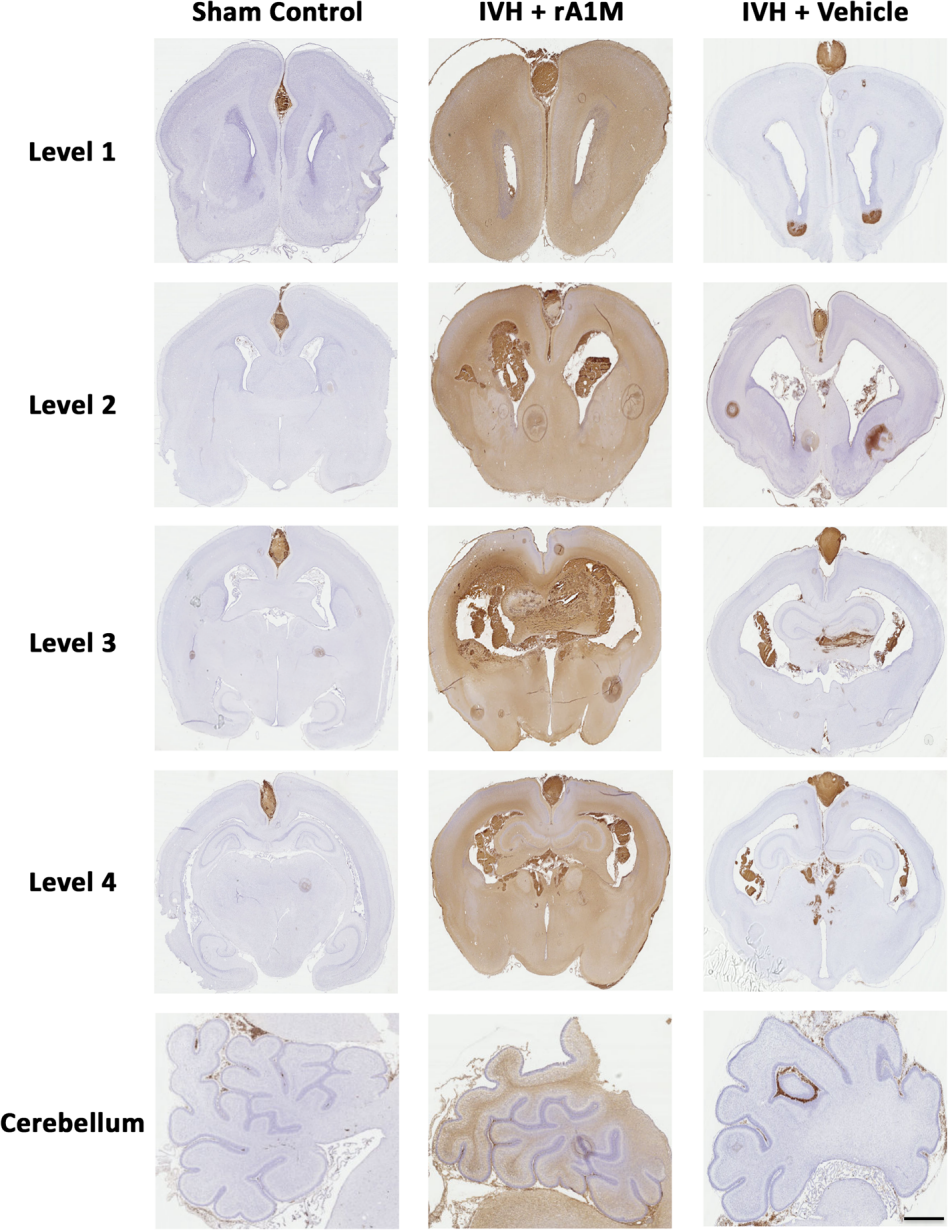
Cerebral and cerebellar A1M distribution following i.c.v. administration of rA1M in rabbit brain following IVH. IHC labeling of A1M was performed to investigate the distribution of i.c.v. administrated rA1M. Rabbit pups with confirmed IVH received i.c.v. injections of either rA1M (IVH + rA1M) or Vehicle (IVH + Vehicle) and were euthanized at 72 h of age followed by saline and freshly prepared 4% PFA perfusion. Brains were prepared and sections from control animals (Sham Control, no bleeding as confirmed with high-frequency ultrasound), IVH + rA1M and IVH + Vehicle animals, at the levels of rostral forebrain (*Level 1*), caudal forebrain (*Level 2*), rostral midbrain (*Level 3*), caudal midbrain (*Level 4*) and *cerebellum* were immunolabeled against A1M as described in the “Materials and methods” section. Microscope analyses were performed on a wide-field Olympus microscope (IX73) and slide scanning were performed on a Hamamatsu NanoZoomer 2.0-HT Digital slide scanner: C10730. Scanning was performed with a 40x magnification lens. Scale bar indicates 5 mm and is applicable for all images

**Fig. 3.**
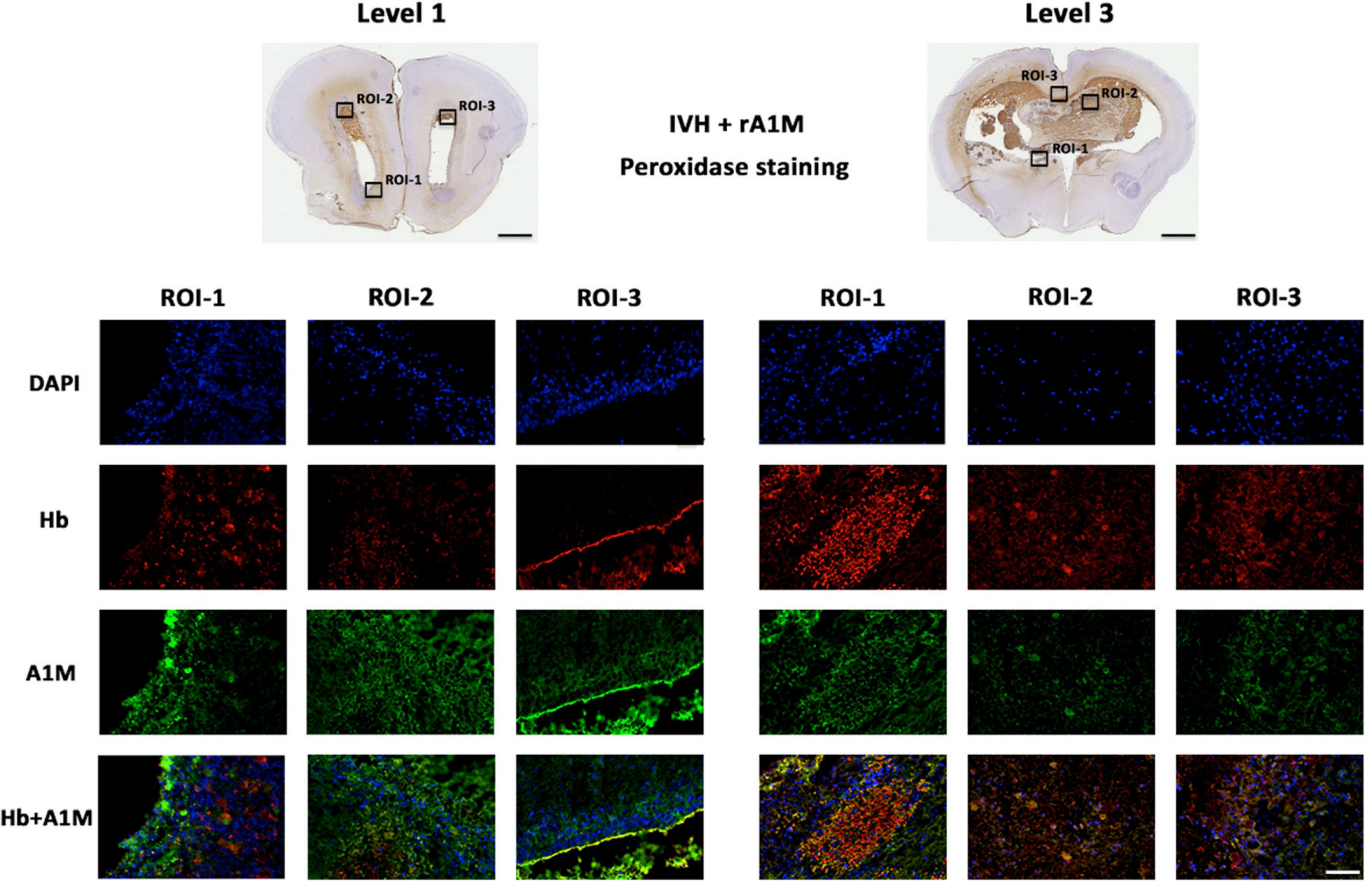
Immunofluorescence labeling of Hb and the i.c.v. administered rA1M. Images represent the double IF-labeled Hb (*red*) and A1M (*green*) together with the nuclear staining DAPI (*blue*), in animals with IVH that were treated with rA1M (IVH + rA1M). Selected ROIs of peroxidase activity from corresponding areas, recorded in parallel sections. Rabbit pups were euthanized at 72 h of age followed by saline and freshly prepared 4% PFA perfusion. Afterwards brains were prepared and sections at the levels of rostral forebrain (*Level 1*) and rostral midbrain (*Level 3*), were immunolabeled for Hb (*red*) and A1M (*green*) as described in the “Materials and methods” section. Fluorescence microscope analyses were performed on a wide-field Olympus microscope (IX73) and slide scanning were performed on a Hamamatsu NanoZoomer 2.0-HT Digital slide scanner: C10730. Scanning was performed with a 40x magnification lens. Images used for illustrations, from ROIs, were grabbed with the viewer software NDP.view2 Viewing software. Scale bar of slide scan images indicates 2.5 mm and in ROI images 25 μm

**Fig. 4.**
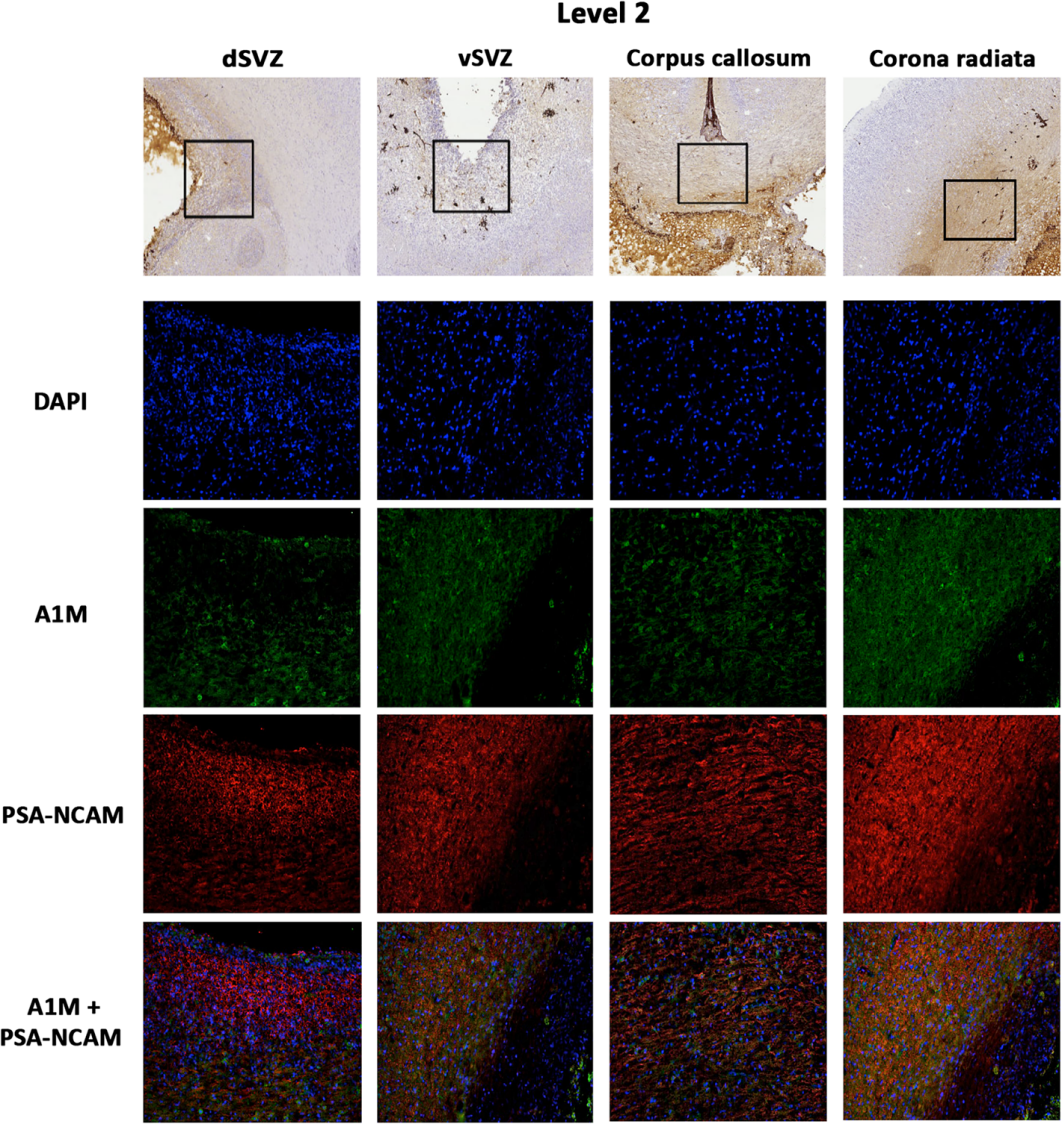
Extensive distribution of i.c.v. administered rA1M within regions of high neuronal plasticity following IVH. The relation between A1M (*green*), and PSA-NCAM (*red*), a marker of areas with high plasticity, together with the nuclear staining DAPI (*blue*), in animals with IVH that were administered with i.c.v. rA1M (IVH + rA1M). Selected ROIs of peroxidase activity from corresponding areas, recorded in parallel sections. Rabbit pups with IVH were euthanized at 72 h of age followed by perfusion with saline and freshly prepared 4% PFA. The brains prepared as described in “Materials and methods” section and a number of neuroanatomically comparable regions of interests, the dorsal subventricular zone (*dSVZ*), ventral SVZ (*vSVZ*), *corpus callosum*, and *corana radiata*, located at the level of caudal forebrain (*Level 2*) were evaluated. Fluorescence microscope analyses were performed on a wide-field Olympus microscope (IX73) and slide scanning was performed on a Hamamatsu NanoZoomer 2.0-HT Digital slide scanner: C10730. Scanning was performed with a 40x magnification lens. Images used for illustrations, from ROIs, were grabbed with the viewer software NDP.view2 Viewing software. Scale bar of slide scan images indicates 2.5 mm and in ROI images 25 μm

To evaluate the presence and distribution of extracellular Hb within the brain, we performed an adapted protocol of the enhanced peroxidase reaction of cryosections [27, 43]. Cryosections, containing the regions of interest (ROI) at the corresponding brain levels (for details of ROI’s and levels see Figs. 2, 3 and 4 and Additional files 1 and 2), were analyzed for peroxidase enzyme activity. Cryosections were air-dried at room temperature (RT) for 20 min, after which, they were then rinsed in PBS, 2 × 10 min. Peroxidase reaction was performed by incubation of the slides in a PBS solution containing di-aminobenzidine (DAB, 25 mg/ml, Sigma-Aldrich, Steinheim, Germany) and hydrogen peroxide (H_2_O_2_, 0.015%, Merck, Darmstadt, Germany) for 10 min at RT. Slides were then rinsed in PBS 3 × 3 min. Sections were counterstained with hematoxylin (Mayers, Histolab) at RT for 2 min, dehydrated, and mounted in Pertex (Histolab). As controls for the peroxidase staining, sections were pre-incubated in 0.03% H_2_O_2_ for 10 min at RT, which totally abolished the endogenous erythrocyte and periventricular tissue peroxidase reaction in non-IVH animals.

### A1M immunohistochemical labeling

IHC labeling of A1M was performed to investigate whether the administrated rA1M could be detected and thereby be used for comparisons between experimental groups and correlated with the peroxidase activity. Cryosections, adjacent to those used for the peroxidase staining (above), were labeled for rA1M by means of IHC.

For IHC labeling of A1M, sections were rinsed in PBS 2 × 3 min and incubated in 0.03% H_2_O_2_ (in PBS, i.e., peroxidase quenching to abolish endogenous peroxidase) for 15 min at RT, followed by rinsing 3 × 3 min in PBS. Slides were then incubated in PBS containing 1% bovine serum albumin (BSA, Sigma-Aldrich) and 0.05% Triton-X 100 (PBS-TX-BSA) for 1 h, at RT. Following 2 × 3 min rinses in PBS, sections were incubated for 2 h at RT with monoclonal antibodies against A1M (23.26, mouse anti-human A1M, in-house developed), diluted in PBS-TX-BSA. Sections were then rinsed in PBS followed by incubation with goat anti-mouse HRP-conjugated secondary antibodies (DAKO Envision+ HRP-polymer kit, Dako North America, Inc., Carpentaria, CA, USA) for 30 min, at RT. Sections were rinsed 3 × 3 min in PBS and were then incubated in PBS solution containing 0.5 mg/ml DAB and 0.015% H_2_O_2_ for 10 min, at RT. Following 3 × 3 min rinses in PBS, sections were counterstained with hematoxylin (Mayers, Histolab), incubated for 2 min at RT, dehydrated in a graded alcohol series ended with 100% xylen, followed by mounting in Pertex (Histolab) and cover-slipped.

### Immunofluorescence labeling

To evaluate the peroxidase staining as representative for Hb-based peroxidase activity and the specificity of rA1M IHC labeling, Hb-immunofluorescence (IF) and rA1M-IF labeling were performed, in adjacent sections as used for peroxidase staining and A1M IHC. In addition, sections were double-IF labeled with the migration and differentiation marker polysialic acid neural cell adhesion molecule (PSA-NCAM).

Brain cryosections from the IVH + rA1M animal group were used for IF labeling. Cryosections adjacent to peroxidase- and rA1M IHC-labeled sections were initially air-dried for 30 min at RT and rinsed in PBS 2 × 3 min. Tissue was then incubated in PBS-TX-BSA for 1 h at RT. Sections were incubated with either separate primary antibody or a mixture of two primary antibodies, for labeling of A1M (chicken anti-human A1M, in-house developed) and Hb (affinity purified goat IgG, GenWay Biotech, San Diego, CA, USA) or PSA-NCAM (Millipore, Temecula, CA, USA), for 16 h at 4 °C.

Sections were then rinsed for 3 × 3 min in PBS and incubated with one fluorophore-conjugated secondary antibody or a mixture of fluorophore-conjugated secondary antibodies made against the species origin of the primary antibodies for 30 min at RT. The secondary antibodies used were affinity-purified Fab2 fragments for multi-labeling, all made in donkey, conjugated with AlexaFluor488, rodamine RX or AlexaFluor647 (Jackson ImmunoResearch Inc, West Grove, PA, USA).

Following 2 × 3 min rinses in PBS, sections were incubated in PBS containing the nuclear stain 4′,6-diamidino-2-phenylindole (DAPI, 0.1 μM in PBS, Molecular Probes, Invitrogen, Rockford, IL, USA), for 20 min, at RT. Sections were rinsed 2 × 3 min in PBS and mounted in “anti-fade solution” (Fluoroshield, Abcam, England or ProLong Gold, Invitrogen).

All animal groups were always processed together in the same immunolabeling experiment.

In every labeling experiment, the primary antibody labeling was tested on parallel sections, in which the primary antibodies were excluded from the labeling protocol. All antibodies used (primary and secondary antibodies) were found to bind to their corresponding antigens, supporting their specific labeling.

### Microscope analysis and image documentation

The detailed distribution of peroxidase staining and rA1M IHC labeling was analyzed and documented with a bright-field microscope (Olympus IX73, Shinjuku, Tokyo, Japan). Slide scanning (Hamamatsu NanoZoomer 2.0ht Model:C900-12, Hamamatsu Sunayama-cho, Nakaku, Hamamatsu City, Japan) was performed of representative individuals of the overall distribution of extracellular Hb and rA1M in the experimental animal groups. Scanned images were used for illustrations.

The fluorescence detection levels (threshold) for each channel (blue: DAPI, green: AF488, red: rodamine RX, and infrared: AF647) were set from control sections lacking primary antibody incubation and from control animal sections (no IVH and/or no rA1M treatment) that had been taken through the complete labeling protocol. Visual analysis and digital imaging of the used fluorophores were performed with the same pre-set detection levels for each channel. The autofluorescence from cell bodies, mainly from RBCs, could be clearly separated from the widely distributed non–RBC extracellular Hb in IVH animals.

### Experimental setup for human plasma A1M (hA1M) functional protection study

Following the brain ultrasound at approx. 6 h of age, pups with IVH (presence of blood within distended lateral ventricles and no sign of parenchymal involvement) were randomized into one of the following groups: IVH + hA1M or IVH + Vehicle. Randomized pups received i.c.v. injections, at approx. 8 h of age, of either hA1M (IVH + hA1M, *n* = 15), purified from human plasma of healthy donors, as described previously [42], or artificial CSF (aCSF, IVH + Vehicle, *n* = 13). The pups received either 25 μl hA1M (9 mg/ml) or 25 μl sterile aCSF using a 27-G Hamilton syringe. For these procedures the rabbit pups were gently fixated on a pre-heated thermostat-controlled platform at 39 °C in a prone position with the probe hand-held by one of the investigators and another investigator performed the needle-insertion under ultrasound guidance. The procedure was performed without sedation of the rabbit pups. The efficacy and accuracy of this method has previously been described [42]. Animals with no detectable IVH at all time-points on cranial ultrasound were used as controls (Sham Control, *n* = 27).

### Tissue collection and processing for mRNA, protein and electron microscopy

Rabbit pups were euthanized at 24 (Sham Control, *n* = 10; IVH + hA1M, *n* = 6; IVH + Vehicle, *n* = 5) and 72 h of age (Sham Control, *n* = 17; IVH + hA1M, *n* = 9; IVH + Vehicle, *n* = 8), and the brains were removed from the skulls and sectioned at the level of the midseptal nucleus. A 1-mm section around the periventricular zone was dissected, snap frozen, and stored at − 80 °C until further mRNA and protein analysis, as described below. For electron microscopy (EM) and EM-immunostaining (EM-IHC), an approx. 1 × 1 × 1 mm piece of periventricular tissue from Sham Controls (*n* = 2), IVH + hA1M (*n* = 2), and IVH + Vehicle (*n* = 2) at 72 h were fixed and prepared as described below. An overview of the study design is shown in Fig. 1b.

### RNA isolation and real-time PCR

Total RNA was isolated from periventricular tissue using the acid guanidinium phenol chloroform method and RNeasy Mini Kit supplied by QIAGEN (Germantown, MD, USA). The OD ratio (optical density at 260 nm/280 nm) of RNA was always higher than 1.9. Reverse transcription was performed according to manufacturer on 0.1-1 μg total RNA using iScript^TM^ cDNA Synthesis Kit (Bio-Rad, CA, USA) and RT^2^ First Strand Kit (QIAGEN). RT^2^ PCR Profiler Array real-time PCR (QIAGEN custom made, Cat no. CAPN11841 and Wound healing, Cat no. 330231 PANZ-121ZA) were used to quantify the mRNA expression of toll-like receptor (TLR)-4, monocyte chemoatractant protein (MCP)-1, interleukin (IL)-1β, IL-6, IL-8, IL1 receptor (R)1, tumor necrosis factor (TNF) α, matrix metalloprotease (MMP) 9, and heme oxygenase (HO)-1. Data were normalized to glyceraldehyde-3-phosphate dehydrogenase (GAPDH, custom made by QIAGEN, Cat no. CAPN11841). The fold change values were calculated by normalizing against Sham Control samples from untreated animals. Data are presented as box plots, displaying medians and 25^th^ and 75^th^ percentiles. Expression was analyzed using RT^2^ SYBR Green Fluor qPCR Mastermix (QIAGEN). Amplification was performed as described by the manufacturer (QIAGEN) for 40 cycles in an iCycler Thermal Cycler (Bio-Rad) and data analyzed using iCycler iQ Optical System Software (Bio-Rad).

### Transmission electron microscopy (TEM)

For ultrathin sectioning, periventricular tissue was fixed for 1 h at RT and then overnight at 4 °C in 2.5% glutaraldehyde (Merck) in 0.15 M sodium cacodylate, pH 7.4 (cacodylate buffer, Sigma-Aldrich). Samples were then washed with cacodylate buffer and post-fixed for 1 h at RT in 1% osmium tetroxide (Agar Scientific Ltd, Stansted Essex, UK) in cacodylate buffer, dehydrated in a graded series of ethanol, and then embedded in Epon 812 (SPI Supplies, West Chester, PA, USA) using acetone (Sigma-Aldrich) as an intermediate solvent. Specimens were sectioned into 50–70 nm ultrathin sections on an LKB ultramicrotome. The ultrathin sections were stained with uranyl acetate (Laurylab, Saint Fons, France) and lead citrate (Laurylab). Immunolabeling of thin sections after antigen unmasking with sodium metaperiodate (Merck) [44] with gold-labeled anti-TNFα (BBInternational, Cardiff, UK) was performed as described previously [45] with the modification that Aurion-BSA (Aurion, Wageningen, The Nethelands) was used as a blocking agent. Specimens were observed in a JEOL JEM 1230 electron microscope operated at 80-kV accelerating voltage. Images were recorded with a Gatan Multiscan 791 CCD camera.

### Statistics

Comparisons between multiple groups were analyzed using ANOVA with post hoc Bonferroni. *P* values < 0.05 were considered significant.

## Results

### Distribution of rA1M following IVH and co-localization within periventricular and cerebellar white matter with extracellular Hb

The distribution of i.c.v. administered rA1M, following IVH, was analyzed in relation to hemorrhage and neuronal plasticity at 72 h of age. Parallel sections were single immunolabeled for A1M, the hemorrhage marker Hb, and double-IF labeled for A1M and Hb or PSA-NCAM.

The propagation of the hemorrhage was characterized as described by Ley et al. [27] by staining all three groups of animals for peroxidase activity of Hb in cerebrum (at four different levels) and cerebellum. In line with Ley et al., we observed a high amount of peroxidase activity throughout the brain from the rostral forebrain (Level 1) through the caudal forebrain (Level 2), rostral midbrain (Level 3), and into the caudal midbrain (Level 4) (Additional file 1). The definition of neuroanatomy and nomenclature used follows that described in the *Atlas of the rabbit brain and spinal cord*, by Shek et al. [46]. Furthermore, peroxidase activity was mainly observed within the ventricle (Level 1 and Level 2), in the subventricular zone (SVZ, Level 1), the choroid plexus (Level 2), the corpus callosum (Level 3), the hippocampus (Level 4), and the thalamus and the subfornical organ (SFO, Level 3 and Level 4) (Additional file 1). Of note, contrary to the study by Agyemang et al. [32], peroxidase activity was detected just on the surface of cerebellar cortex in IVH animals and not in the white matter (Additional file 1). This may be due to the difference in methodology employed in the detection of the cell-free Hb, peroxidase activity in the present study as compared to IF labeling in the previous study.

Furthermore, Hb immunolabeling was performed to confirm that peroxidase activity was related to the presence of Hb showing the existence of both in corresponding brain periventricular white matter regions and fiber tracts (Level 1, ROI-1-3; Level 3, ROI-1-3) (Fig. 3).

The IHC staining for A1M showed an extensive distribution within brain and cerebellum in all IVH animals that received i.c.v. rA1M injections, whereas the IVH + Vehicle and Sham Control animals only displayed peroxidase activity corresponding to the hemorrhage (Fig. 2). A1M immunoreactivity was, to a varying degree, distributed in several brain regions, such as in the septum, striatum, corona radiata, corpus callosum, lateral ventricular zones, and in the cortex (Additional file 2; Levels 1–4). Similar to extracellular Hb, A1M labeling was profound along the ventricular walls, in the neurogenetic brain nuclei and in fiber tracts in the parenchyma (Fig. 3; Level 1, ROI-1-3; Level 3, ROI-1-3).

Notably, characterization of IF double-labeling for A1M and Hb was characterized by co-existence and even co-localization at the cellular level within periventricular white matter, fiber tracts, thalamic region, corpus callosum, corona radiata, and hippocampus (Fig. 3; Level 1, ROI 1–3; Level 3, ROI 1–3).

In the view of such a wide distribution of rA1M and co-existence with extracellular Hb within highly differentiated zones and axonal fiber tracts, we explored further the relationship to staining with PSA–NCAM, a marker of neuronal plasticity, including axonal growth, cell migration, synaptic plasticity, neuronal-glial plasticity, and neurogenesis [47]. Double labeling of A1M and PSA-NCAM showed a pronounced co-existence within periventricular and cerebellar areas with high plasticity (white matter, subventricular zone, corpus callosum, corona radiate, thalamocortical projection, Fig. 4).

### hA1M reduced the IVH-induced structural damage and mitochondrial hypertrophy

**Fig. 5:**
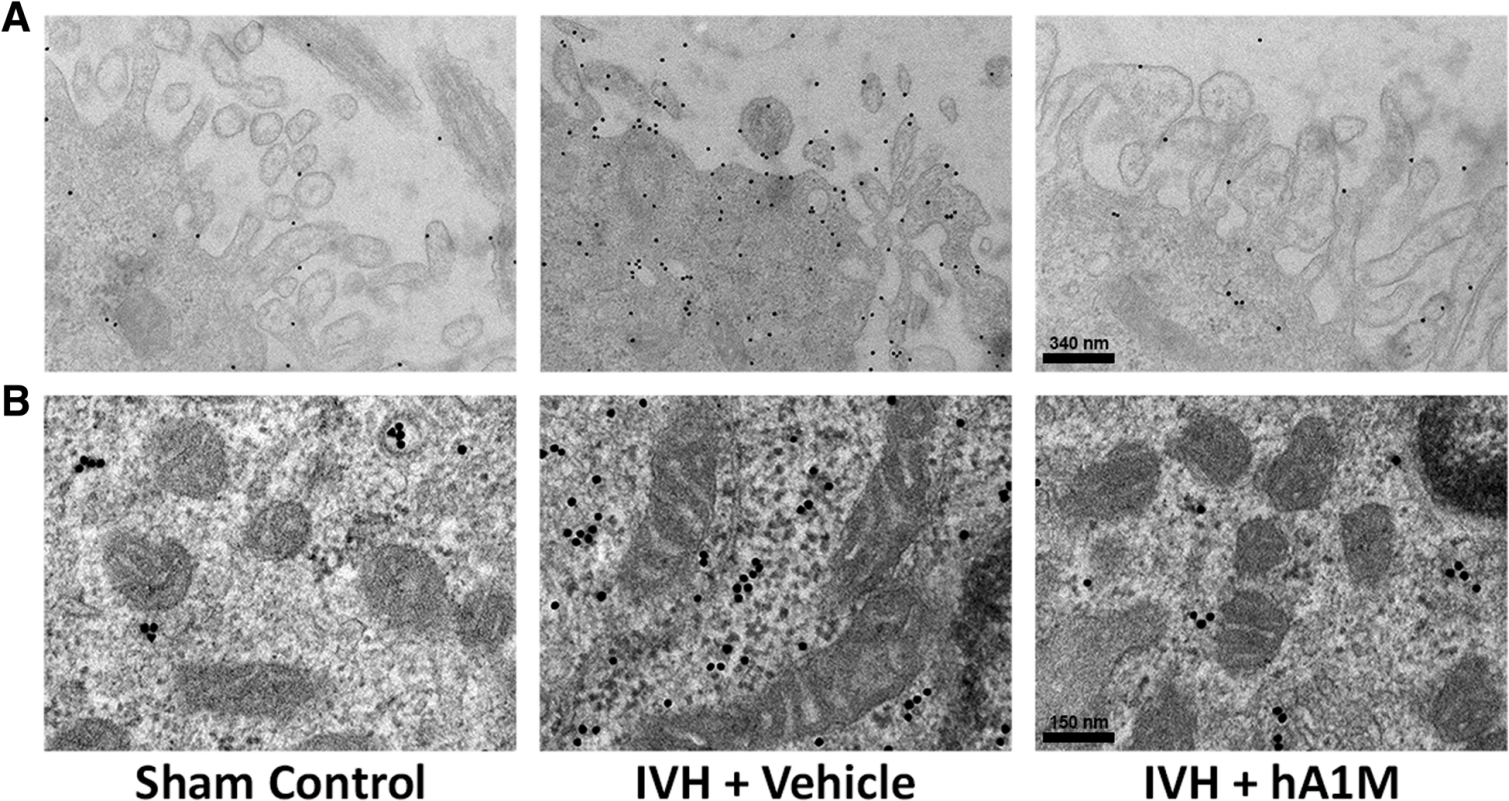
hA1M reduces structural damage and mitochondrial hypertrophy. Rabbit pups with confirmed IVH, injected i.c.v. with hA1M (IVH + hA1M) or Vehicle (IVH + Vehicle) or Sham Control (no bleeding as confirmed with high-frequency ultrasound), were euthanized at 72 h of age and the brains were removed from the skulls, sectioned at the level of the midseptal nucleus and choroid plexus was fixed and prepared as described in the “Materials and methods” section and observed in a Jeol JEM 1230 electron microscope. Scale in top panel (**a**) indicates 340 nm and in lower panel (**b**) 150 nm

Structural and cellular damage was investigated following IVH in preterm rabbit pups at 72 h of age. Electron micrographs obtained from the ependymal epithelium of the periventricular lining, of pups with IVH, showed substantial ultrastructural damage, e.g. disintegration of the microvilli of the ependymal, cell mitochondrion perturbations marked by the formation of swollen mitochondria and a simultaneous marked upregulation in TNFα expression (IVH + Vehicle, Fig. 5). Intracerebroventricular administration of hA1M (IVH + hA1M) was associated with reduced ultrastructural damage, seen as minor structural changes in microvilli, inhibition of mitochondrial swelling, and blocked upregulation of TNFα expression, making them comparable to the Sham Control.

### hA1M reduced IVH-induced up-regulation of proinflammatory markers, cellular response, oxidative stress and matrix degradation proteins

**Fig. 6:**
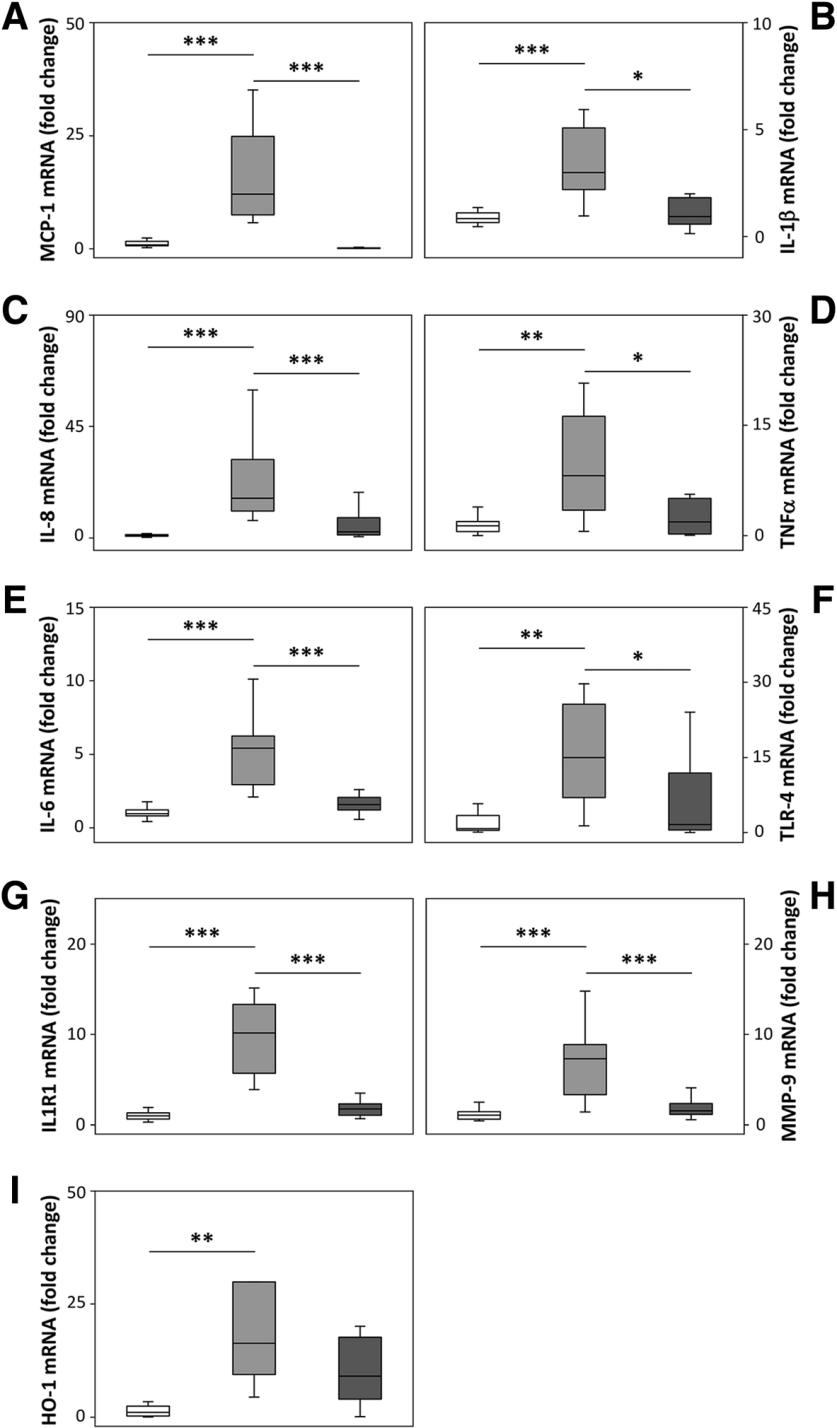
hA1M reduces inflammatory response, cellular activation, oxidative stress and matrix degradation proteins. Rabbit pups with confirmed IVH, i.c.v. injected with hA1M (IVH + hA1M, *dark grey bars*; *n* = 9) or Vehicle (IVH + Vehicle, *grey bars*; *n* = 8) or Sham Control (*white bars*; *n* = 17; no bleeding as confirmed with high-frequency ultrasound) were euthanized at 72 h of age and the brains were removed from the skulls and a piece of periventricular tissue was carefully removed from the lateral ventricles, snap frozen, and the mRNA expressions of MCP-1 (**a**), IL-1β (**b**), IL-8 (**c**), TNFα (**d**), IL-6 (**e**), TLR-4 (**f**), IL1R1 (**g**), MMP-9 (**h**) and HO-1 (**i**) were subsequently analyzed with real-time PCR, as described in the “Materials and methods” section. mRNA expressions for the respective genes were normalized against those of GAPDH and are given as fold change. The fold change values were calculated by normalizing against samples from control animal (Sham Control). Results are presented as box plots displaying medians and 25^th^ and 75^th^ percentiles. Differences between IVH + hA1M vs. IVH + Vehicle and IVH + Vehicle vs. Sham Control at 72 h were analyzed using ANOVA post hoc Bonferroni. *P < 0.05, **< 0.01, ***< 0.001

Analysis of mRNA expression in periventricular tissue displayed a significant upregulation of proinflammatory markers MCP-1, IL1β, IL-8, TNFα, and IL-6 at 72 h following IVH (IVH + Vehicle vs. Sham Control) (Fig. 6a–e). Additionally, all genes displayed a marked upregulation at 24 h, although only TNFα was found to be significantly increased (not shown). Intracerebroventricular administration of hA1M significantly reduced the mRNA expression of all measured proinflammatory markers (IVH + hA1M vs. IVH + Vehicle), to levels comparable to the control animals (Sham Control), at both 24 (not shown) and 72 h (Fig. 6a–e).

Analysis of mRNA expression of the receptor-related signaling genes TLR-4 and IL1R1 also showed a significant upregulation at 72 h following IVH (IVH + Vehicle) that was significantly reduced following i.c.v. administration of hA1M (IVH + hA1M) to levels comparable to those of the control animals (Sham Control) (Fig. 6f, g).

To further investigate the event of tissue injury and remodeling in IVH, we analyzed mRNA expression of the extracellular matrix degradation protein, MMP-9. IVH (IVH + Vehicle) caused a marked upregulation of MMP-9, as compared to the control (Sham Control) animals, at 72 h with a significantly reduction following i.c.v. administration of hA1M (IVH + hA1M) (Fig. 6h).

Analysis of the major heme-degrading stress protein HO-1 displayed a marked up-regulation in the IVH + Vehicle group, as compared to control animals (Sham Control), at 72 h. An appreciable reduction, however not significant, was observed following hA1M (IVH + hA1M) administration (Fig. 6i).

## Discussion

In this study, we show that following i.c.v. administration of rA1M in preterm rabbit pups with IVH, there is a wide distribution of rA1M in periventricular and cerebellar regions with high plasticity (white matter, subventricular zone, corpus callosum, corona radiata, thalamocortical projection; as shown by PSA-NCAM) coupled with a high co-existence of deposited extracellular Hb. We also show that i.c.v. administration of hA1M confers an early functional protection in periventricular brain tissue following IVH.

Following preterm IVH, there is a deposition of extravasated blood the intraventricular space. This deposition is followed by a migration of RBCs and extracellular Hb to the surrounding periventricular tissue and to remote regions of the cerebellar white matter [27, 32]. Due to the potential cytotoxic nature of extracellular Hb this distribution becomes highly relevant in understanding and characterizing the damages to the immature brain following IVH. Extracellular Hb may cause cerebral damage in a number of different ways. Following hemolysis, extracellular Hb undergoes a series of biochemical changes leading to the sequential formation of metHb, heme, and free iron, all of which can generate free radicals and ROS. These serve to increase the redox activity of the extracellular environment leading to a pro-oxidative challenge to the brain cells. Indeed, redox-related effects resulting from heme and iron overload have been reported to cause cerebral damage following IVH [48, 49], and administration of desferamine, an iron chelator, has been shown to attenuate the development of hydrocephalus and brain damage in a rodent model of neonatal GM hemorrhage [50]. In addition to its redox-related effects, heme has also been described to act as a damage-associated molecular pattern (DAMP) molecule triggering TLR-mediated proinflammatory damaging pathways [51–53]. Furthermore, Agyemang and colleagues have shown the causal involvement of extracellular Hb in compromised cerebellar neuronal progenitor proliferation and delayed Purkinje cell maturation following IVH in preterm rabbit pups [32].

A1M has been shown to have strong cell- and tissue-protective effects utilizing a multifaceted palette of mechanisms, including detoxification of free radicals and ROS, capturing of free heme groups, inducing tissue repair mechanisms, and protecting the mitochondria. Furthermore, rA1M has been described to constitute a potential therapeutic drug candidate in treatment or prophylaxis of diseases or conditions that are associated with pathological oxidative stress [36, 39, 54–56].

The brain is reported to have a minimal production and systemic distribution of endogenous A1M, reflected in the trace quantities of the protein present in CSF as compared to other body fluids, CSF (0.0423 mg/l), serum (44.2 mg/l), synovial fluid (20.8 mg/l), ascites (28.7 mg/l), pleural effusion (21.5 mg/l), and amniotic fluid (0.0027 mg/l) [57]. Interestingly, an increase in the concentration of endogenous A1M in CSF following cerebral hemorrhage has been documented in adult human patients. The increase was associated with serum infiltration as a result of blood-brain barrier disruption, but a local production could not be excluded [58].

Congruent with the described pathophysiological mechanisms for brain damage following IVH, exogenously administered A1M makes a very interesting treatment alternative hence the premise for the current study.

The aim of the biodistribution studies, was to investigate whether exogenously administered A1M actually reaches the anatomical regions of the brain which are affected following IVH. The results of this study showed a wide distribution of i.c.v. administered rA1M within the immature brain following IVH. Indeed, immunolabeling for A1M demonstrated a presence of exogenous rA1M along periventricular areas with an increased amount within white matter tracts (Fig. 2). Cell adhesion molecules (CAMs) are important for normal development and maintenance of the architecture of the central nervous system (CNS). Several molecule families, as integrin, cadherin, semaphorin, and immunoglobulin, are involved in CNS morphogenesis and plasticity [59–61]. NCAM is the most expressed CAM on the cell surface in CNS, and NCAM is responsible for establishing cell-cell connection [47]. PSA-NCAM has a wide distribution in the embryonic and early postnatal period and is considered a marker for both immature neural precursor cells as well as associated with glial precursors [62, 63]. In this study, PSA-NCAM staining in these regions was positive, indicating a high plasticity and reduced cellular density, characteristics that allow for a certain degree of diffusion of molecules. Interestingly, double immunolabeling for PSA-NCAM and A1M displayed a high co-existence (Fig. 4).

Furthermore, it appears that rA1M distribution follows a similar distribution pattern as extracellular Hb, as shown previously by Ley et al. [27] and confirmed by the current study. In fact, IF labeling of A1M and Hb displayed a high co-existence and indications of co-localization (Fig. 3).

The mechanisms enabling the diffusion of extracellular Hb and rA1M are not known. Diffusion-MRI studies have shown diffusion of water molecules not only within damaged brain tissue [64, 65] but also in relation to brain activity [66, 67]. Interestingly, it has been shown that diffusion of water is faster in the white matter fiber tracts, which probably depends on gradient pulse direction [64, 68]. The finding that both extracellular Hb and rA1M have a wide distribution along white matter tracts following IVH in preterm rabbit pups indicates such diffusion also of larger molecules [27]. In fact, rA1M was found not only in cerebral white matter tracts but also in cerebellar white matter (Fig. 2), a finding in line with the study by Agyemang et al. [32].

In addition, it has been shown in human preterm infants that PHVD leads to mechanical compression of surrounding periventricular white matter with subsequent alteration of neurophysiological characteristics [69]. Recently, Brouwer and colleagues showed altered diffusion characteristics on brain MRI in preterm infants with PHVD [70] suggesting a disruption of white matter integrity. Of note, in our preterm rabbit pup IVH model, unlike in human infants, PHVD develops rapidly within 72 h following glycerol injection [42]. Thus, a possible loss of white matter integrity, in conjunction with the widespread presence of rA1M within white matter tracts following i.c.v. administration, could indicate a permissive diffusion towards a concentration gradient following IVH.

At present, GM-IVH is neither preventable nor treatable. A significant proportion of extremely premature infants develop GM-IVH and even a small bleeding can cause impaired neurocognitive outcome. In this study, we evaluated early functional effects of i.c.v. administration hA1M in rabbit pups following detection of IVH. Analysis of structural integrity, using EM-IHC, displayed a preserved structure and an arrest of the fusion of adjacent mitochondria in megamitochondria formation. Furthermore, hA1M administration significantly reduced cellular activation, inflammatory response, and tissue injury, suggesting that administration of hA1M blocks the toxic reactions of extracellular Hb-metabolites. The mechanisms whereby administrated hA1M offers protection following IVH are not known. However, hA1M, as well as rA1M, has previously been shown to display in vitro heme-binding, radical-binding, and reductase properties [35, 40, 41]. The results thus suggest that the antioxidation properties of human A1M can ameliorate the early IVH-symptoms, suggesting that heme-induced oxidative stress and inflammatory response are indeed important etiological factors. Oxidative stress and inflammation have been shown to be closely related, and the use of antioxidant therapeutic strategies has been explored in several types of cerebral hemorrhage [71]. Furthermore, Vinukondo and colleagues showed that the use of the TNFα inhibitors, celecoxib and etanercept, reduced cerebral white matter impairment following IVH [72].

Although this study outlines important findings, it is clear that further studies are needed to evaluate the protective effects of hA1M/rA1M following IVH. Although extensive evaluation of functionally relevant parameters was performed, these were evaluated on mRNA level only and the corresponding changes in protein levels were not determined. Importantly, changes in mRNA levels are not always reflected in parallel protein changes. In the present study, the protein levels in relevant tissue extracts were unfortunately not possible to analyze due to a limitation in available material.

Ongoing studies aim to investigate if the observed short-term effects of hA1M/rA1M following IVH result in improved short- and long-term neurodevelopmental and behavioral outcome.

## Conclusions

In this study, we showed that following i.c.v. administration, there is a similar brain distribution pattern of rA1M as of the endogenous extracellular Hb in preterm IVH rabbit pups. The distribution extended to areas of high plasticity within white matter of the periventricular tissue and cerebellum. Additionally, we showed a protective role exhibited by hA1M on periventricular tissue following IVH in the preterm rabbit pup. Taken together, this study suggests rA1M/hA1M as a potential neuroprotective intervention against brain damage following preterm IVH.

## Additional files

Additional file 1: **Supplementary figure 1 showing the distribution of Hb following IVH in preterm rabbit pup.** The presence of Hb following IVH was characterized within the brain utilizing the inherent peroxidase activity of Hb. Rabbit pups with confirmed IVH, i.c.v. injected with rA1M (**IVH + rA1M**) or Vehicle (**IVH + Vehicle**) or **Sham Controls** were euthanized at 72 hours of age followed by saline and freshly prepared 4% PFA perfusion. The brains prepared as described in Materials and Methods and a number of neuroanatomically comparable regions of interests, located at the levels of rostral forebrain (**Level 1**), caudal forebrain (**Level 2**), rostral midbrain (**Level 3**), caudal midbrain (**Level 4**) and **cerebellum**, were stained for peroxidase activity of Hb as described in the Materials and Methods. Microscope analyses were performed on a wide-field Olympus microscope (IX73) and slide scanning were performed on a Hamamatsu NanoZoomer 2.0-HT Digital slide scanner: C10730. Scanning was performed with a 40x magnification lens. Images used for illustrations, were grabbed with the viewer software NDP.view2 Viewing software. Scale bar of slide scan image indicate 2.5 mm and of grabbed images indicate 500 μm. (TIF 5824 kb)

Additional file 2: **Supplementary figure 2 showing the distribution of A1M following i.c.v. administration of rA1M in preterm rabbit pups with IVH.** IHC labeling of A1M was performed to investigate the distribution of i.c.v. administrated rA1M. To correlate the rA1M distribution with that of extracellular Hb (peroxidase activity), cryosections adjacent to those used for the peroxidase staining, were immunolabeled for A1M as described in the Materials and Methods Section. Rabbit pups with confirmed IVH received i.c.v. injections of either rA1M (**IVH + rA1M**) or Vehicle (**IVH + Vehicle**) and were euthanized at 72 hours of age followed by saline and freshly prepared 4% PFA perfusion. Brains were prepared and a number of neuroanatomically comparable regions of interests, located at the levels of rostral forebrain (**Level 1**), caudal forebrain (**Level 2**), rostral midbrain (**Level 3**) and caudal midbrain (**Level 4**), were stained for A1M as described in the Materials and Methods. Microscope analyses were performed on a wide-field Olympus microscope (IX73) and slide scanning were performed on a Hamamatsu NanoZoomer 2.0-HT Digital slide scanner: C10730. Scanning was performed with a 40x magnification lens. Images used for illustrations, from ROIs, were grabbed with the viewer software NDP.view2 Viewing software. Scale bar of slide scan image indicate 2.5 mm and of ROI images indicate 500 μm. (TIF 6323 kb)

## Abbreviations

A1M: α_1_-microglobulin
BSA: Bovine serum albumin
CAM: Cell adhesion molecule
CNS: Central nervous system
CSF: Cerebrospinal fluid
DAB: Di-aminobenzidine
DAMP: Damage-associated molecular patterns
DAPI: 4′,6-diamidino-2-phenylindole
DNA: Deoxyribonucleic acid
EM: Electron microscopy
GAPDH: Glyceraldehyde-3-phosphate dehydrogenase
GM: Germinal matrix
GM-IVH: Germinal matrix intraventricular hemorrhage
H_2_O_2_: Hydrogen peroxide
hA1M: Human A1M purified from plasma
Hb: Hemoglobin
HO-1: Heme oxygenase-1
I.c.v.: Intracerebroventricular
IF: Immunofluorescence
IHC: Immunohistochemistry
IL: Interleukin
IL1R: Interleukin-1 receptor
IVH: Intraventricular hemorrhage
MCP-1: Monocyte chemoatractant protein -1
MetHb: Methemoglobin, ferric hemoglobin
MMP-9: Matrix metalloprotease
MRI: Magnetic resonance imaging
OD: Optical density
OxyHb: Oxyhemoglobin, ferrous hemoglobin
PBS: Phosphate buffer saline
PFA: Paraformaldehyde
PHVD: Post-hemorrhagic ventricular dilatation
PSA-NCAM: Polysialic acid neural cell adhesion molecule
rA1M: Recombinant human A1M
RBC: Red blood cells
ROI: Regions of interest
ROS: Reactive oxygen species
RT: Room temperature
TEM: Transmission electron microscopy
TLR-4: Toll-like receptor
TNF: Tumor necrosis factor
VLBW: Very low birth weight
