# Supplementary figures and images for: The heme and radical scavenger α1-microglobulin (A1M) confers early protection of the immature brain following preterm intraventricular hemorrhage
Source: J Neuroinflammation. 2019 Jun 7;16:122. doi: 10.1186/s12974-019-1486-4 (PMC6554963; doi:10.1186/s12974-019-1486-4)

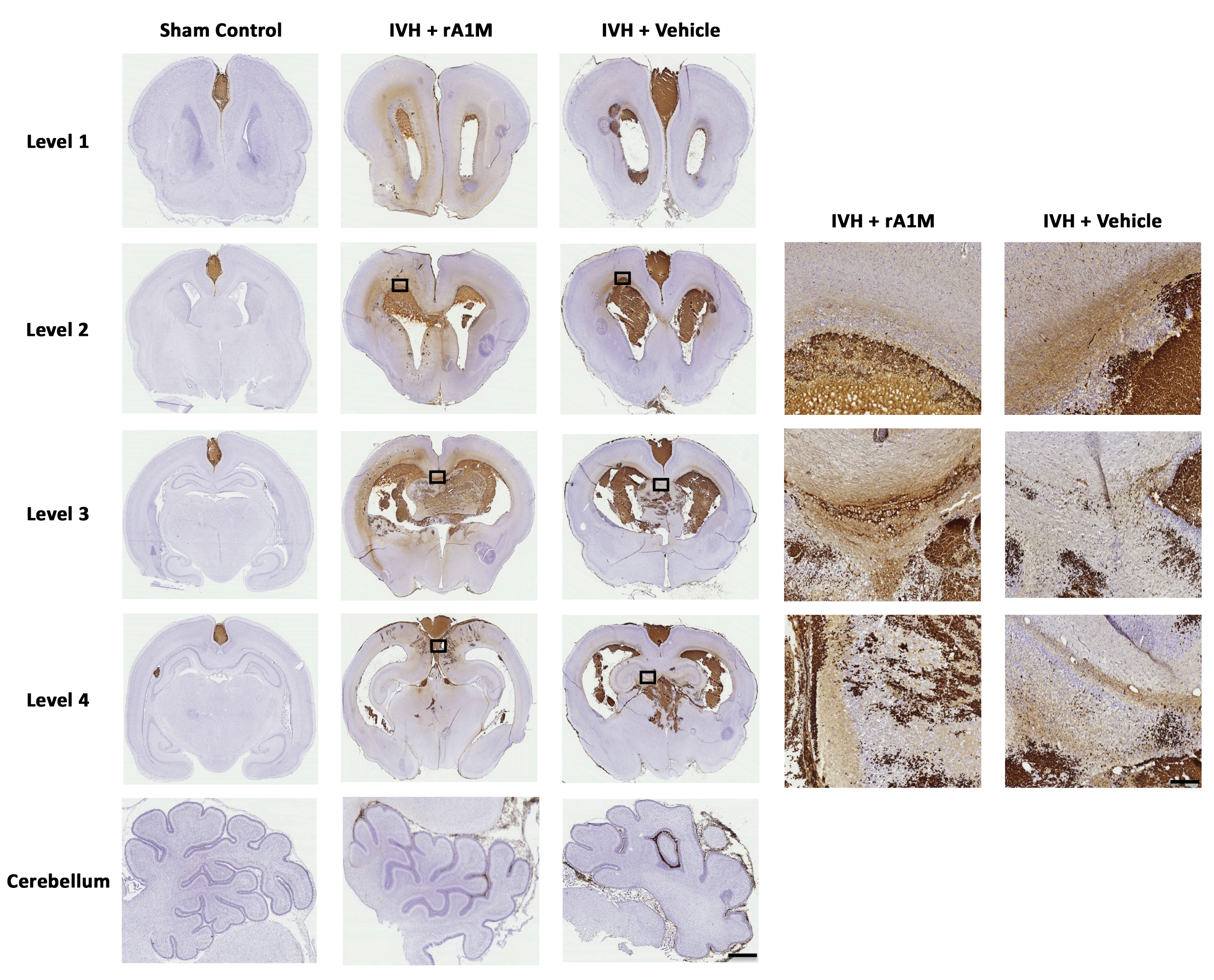

Supplement: Supplementary file 1 — Supplementary figure 1 showing the distribution of Hb following IVH in preterm rabbit pup. The presence of Hb following IVH was characterized within the brain utilizing the inherent peroxidase activity of Hb. Rabbit pups with confirmed IVH, i.c.v. injected with rA1M (IVH + rA1M) or Vehicle (IVH + Vehicle) or Sham Controls were euthanized at 72 hours of age followed by saline and freshly prepared 4% PFA perfusion. The brains prepared as described in Materials and Methods and a number of neuroanatomically comparable regions of interests, located at the levels of rostral forebrain (Level 1), caudal forebrain (Level 2), rostral midbrain (Level 3), caudal midbrain (Level 4) and cerebellum, were stained for peroxidase activity of Hb as described in the Materials and Methods. Microscope analyses were performed on a wide-field Olympus microscope (IX73) and slide scanning were performed on a Hamamatsu NanoZoomer 2.0-HT Digital slide scanner: C10730. Scanning was performed with a 40x magnification lens. Images used for illustrations, were grabbed with the viewer software NDP.view2 Viewing software. Scale bar of slide scan image indicate 2.5 mm and of grabbed images indicate 500 μm. (TIF 5824 kb) [file 12974_2019_1486_MOESM1_ESM.tif]

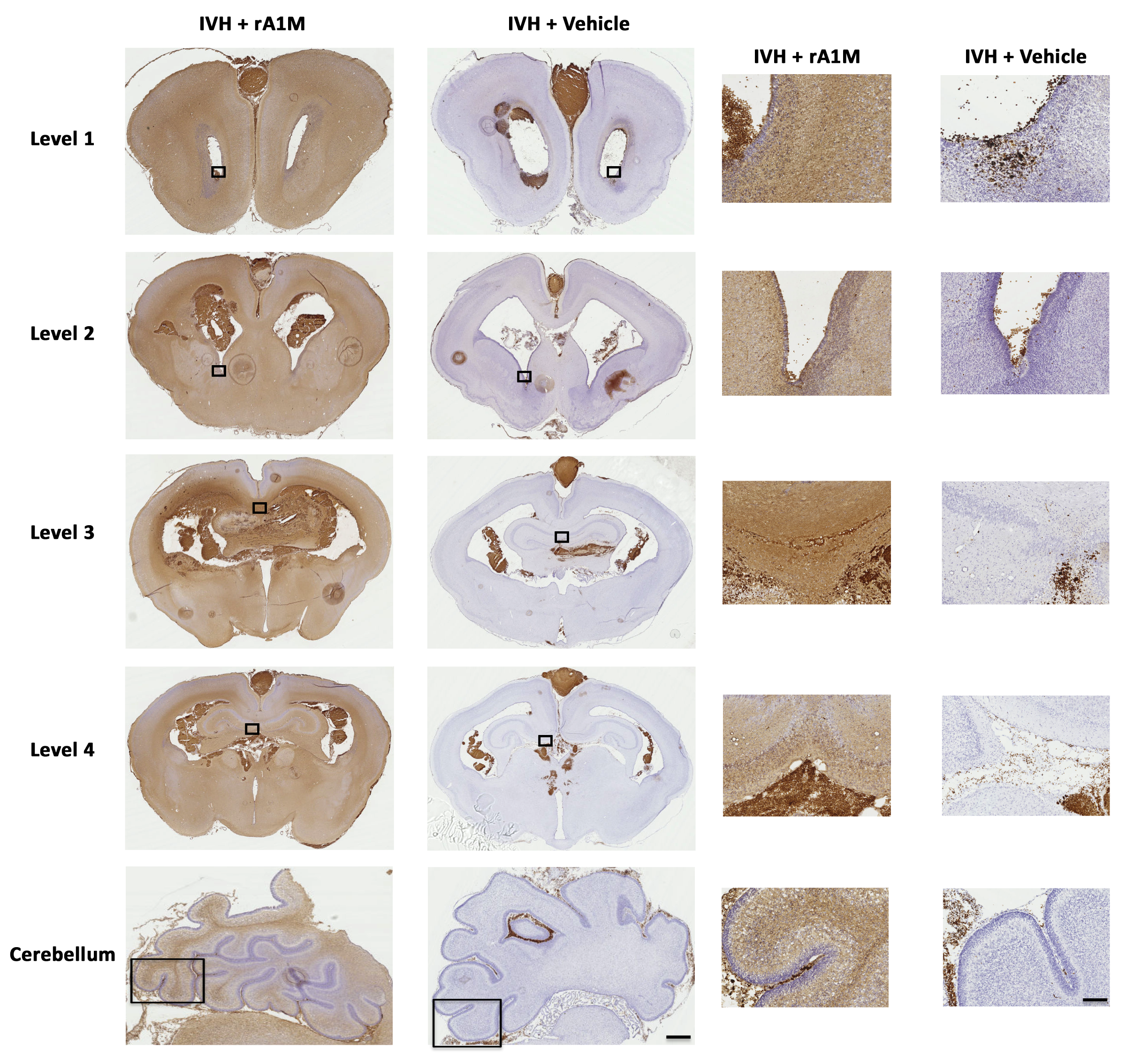

Supplement: Supplementary file 2 — Supplementary figure 2 showing the distribution of A1M following i.c.v. administration of rA1M in preterm rabbit pups with IVH. IHC labeling of A1M was performed to investigate the distribution of i.c.v. administrated rA1M. To correlate the rA1M distribution with that of extracellular Hb (peroxidase activity), cryosections adjacent to those used for the peroxidase staining, were immunolabeled for A1M as described in the Materials and Methods Section. Rabbit pups with confirmed IVH received i.c.v. injections of either rA1M (IVH + rA1M) or Vehicle (IVH + Vehicle) and were euthanized at 72 hours of age followed by saline and freshly prepared 4% PFA perfusion. Brains were prepared and a number of neuroanatomically comparable regions of interests, located at the levels of rostral forebrain (Level 1), caudal forebrain (Level 2), rostral midbrain (Level 3) and caudal midbrain (Level 4), were stained for A1M as described in the Materials and Methods. Microscope analyses were performed on a wide-field Olympus microscope (IX73) and slide scanning were performed on a Hamamatsu NanoZoomer 2.0-HT Digital slide scanner: C10730. Scanning was performed with a 40x magnification lens. Images used for illustrations, from ROIs, were grabbed with the viewer software NDP.view2 Viewing software. Scale bar of slide scan image indicate 2.5 mm and of ROI images indicate 500 μm. (TIF 6323 kb) [file 12974_2019_1486_MOESM2_ESM.tif]
